# Extreme Levels of Noise Constitute a Key Neuromuscular Deficit in the Elderly

**DOI:** 10.1371/journal.pone.0048449

**Published:** 2012-11-06

**Authors:** Navrag B. Singh, Niklas König, Adamantios Arampatzis, Markus O. Heller, William R. Taylor

**Affiliations:** 1 Julius Wolff Institute, Center for Sports Science and Sports Medicine Berlin (CSSB), Charité – Universitätsmedizin Berlin, Berlin, Germany; 2 Department of Training and Movement Science, Center for Sports Science and Sports Medicine Berlin (CSSB), Humboldt-Universität zu Berlin, Berlin, Germany; University of South Australia, Australia

## Abstract

Fluctuations during isometric force production tasks occur due to the inability of musculature to generate purely constant submaximal forces and are considered to be an estimation of neuromuscular noise. The human sensori-motor system regulates complex interactions between multiple afferent and efferent systems, which results in variability during functional task performance. Since muscles are the only active component of the motor system, it therefore seems reasonable that neuromuscular noise plays a key role in governing variability during both standing and walking. Seventy elderly women (including 34 fallers) performed multiple repetitions of isometric force production, quiet standing and walking tasks. No relationship between neuromuscular noise and functional task performance was observed in either the faller or the non-faller cohorts. When classified into groups with either nominal (group NOM, 25^th^ –75^th^ percentile) or extreme (either too high or too low, group EXT) levels of neuromuscular noise, group NOM demonstrated a clear association (r^2^>0.23, p<0.05) between neuromuscular noise and variability during task performance. On the other hand, group EXT demonstrated no such relationship, but also tended to walk slower, and had lower stride lengths, as well as lower isometric strength. These results suggest that neuromuscular noise is related to the quality of both static and dynamic functional task performance, but also that extreme levels of neuromuscular noise constitute a key neuromuscular deficit in the elderly.

## Introduction

The order of recruitment as well as the variability in the firing of multiple motor units during voluntary isometric submaximal muscular contraction results in an inability of the musculature to generate constant forces, causing oscillations or fluctuations in the resulting force output [1]–[7]. The level of force fluctuation is thus modulated by physiological parameters such as the number, size and discharge rates of the motorneurons, as well as muscle fibre type etc. [1]. Due to these physiological characteristics, the level of fluctuation during isometric force generation is considered to be an approximation of the amount of *neuromuscular noise*, considered to be disturbances that obscure the desired output of the sensorimotor system [7]–[9]. Furthermore, neuromuscular noise is known to be proportional to the force output [10]. The variability of kinetics and kinematics are therefore dependent upon the requirements of the task, such that e.g. final location accuracy is reduced with increasing trajectory speed [10], [11].

Since this variability within the generated force is known to influence the intended movement trajectory [4], [11]–[13], the presence of neuromuscular noise is thought to affect the trial-to-trial repetitions of a task [1], [10]. The effective regulation of neuromuscular noise is therefore a prerequisite for continuous or repetitive functional task performance, such as standing (postural sway) and walking (gait variability) [11], [14]–[16]. The level of force fluctuations during muscular contractions [1], [2], [4], [17] thus needs to be accounted for in order to optimise kinetics and kinematics during task performance, and this is achieved through feedback mechanisms within the sensori-motor system [1], [11], [15], [16], [18]–[21]. It is reasonable that variability during repetitive tasks provides a measure of an individual’s static or dynamic task performance, and could be used to assess the limitations of their system. The quantification of variability during task performance has therefore become a target for evaluating the human sensori-motor system [4], [16], [22]–[28].

Variability during task performance is omnipresent and does not necessarily suggest motor related pathology [14], [28]. In fact, variability is an indicator of redundancy within the system that allows performance adaptation, and is therefore thought to be necessary for optimised task learning [29], [30]. On the other hand, excessive variability may indicate that the system is operating closer to the limits of ability, expressed as stability during balance and gait [14], [25]–[27], [31], [32]. Here, in order to maintain stability, the centre of mass must be effectively maintained within the base of support. A stable system during standing would therefore either stay in or return to a state of equilibrium after being perturbed [23], [33]. In a similar manner, during dynamic conditions e.g. walking, a stable system would remain in a state of uniform motion, maintained by suitable foot placement. Systems with higher levels of variability would thus require greater error correction in order to maintain the centre of mass within the base of support. In fact, higher levels of variability during standing and walking, have been observed in individuals that are at increased risk of falling, as well as in those who suffer from motor related pathologies [22], [27], [32]. Until now, variability during static or dynamic functional tasks has been viewed independently [18], [21], [23], [25], [34], [35], but it seems plausible that variability during specific tasks results from core system characteristics such as neuromuscular noise, together with local functional ability, including strength, coordination and training of the specific musculature. Since muscles are the only active component of the motor system, it seems reasonable that neuromuscular noise in the lower extremities could explain variability during upright (standing posture) tasks including standing and walking. An understanding of the contribution of force fluctuations, as a measure of neuromuscular noise, on balance and walking performance in elderly populations would not only help to provide an understanding of the aetiology of postural sway and gait variability, but might also aid in the early diagnosis of motor related pathologies, particularly in individuals with balance and gait deficits. The aim of this study was therefore to investigate the relationship between neuromuscular noise and static and dynamic task variability among elderly healthy and faller cohorts.

## Methods

### Participants

Within a larger study examining the risk of fracture in the elderly (EU VPHOP FP7–223864), we examined ninety elderly participants from the local community. Of these 90, a number of subjects who reported conditions of arthritis, artificial joints, diabetes and/or herniated vertebral discs were excluded from the study cohort for this analysis. As a result, seventy elderly women (34 with at least 1 fall within the previous 12 months - “faller”; 36 healthy controls – “non-faller”) undertook the experimental protocol. All participants provided written informed consent and the experiments were approved by the local ethics committee. Both groups were homogenous in terms of age, weight and height with a mean (± SD) of: 69.8 (±4.8) years, 69.7 (±10.2) kg and 163.1 (±6.6) cm for elderly fallers, and 69.2 (±4.6) years, 67.7 (±10.7) kg and 162.1 (±6.0) cm for elderly non-faller cohorts respectively.

### Experimental Design and Procedures

Within each test session, participants performed a minimum of 3 test repetitions or trials to examine force fluctuations, postural sway, and walking variability in separate sessions conducted on the same day. Force fluctuations were measured on the right limb during isometric knee extension and isometric ankle plantar-flexion. Postural sway was measured during quiet standing in a biped position with eyes open. Gait assessment was performed at preferred walking speed.

### Force Fluctuation Measurements

In this study, force fluctuations were considered an indirect measure of neuromuscular noise, and were assessed in the knee extensors and ankle plantarflexors. Briefly, participants were seated in a standardised position in a Biodex 3 Pro dynamometer (Biodex Medical Systems Inc., USA) [17]. Before each measurement the flexion/extension rotation axis of the tested joint was aligned with the rotational axis of the dynamometer. Knee extension measurements were then conducted with the knee flexed at 90 degrees, while for ankle measurements, the knee was fully extended with 10° of plantarflexion at the ankle. Prior to the start of each force fluctuation session, maximum voluntary isometric contractions (MVICs) were obtained by providing standardised instructions and verbal encouragement, trying to reach peak exertion 2–3 s after the start of the trial. MVICs, which lasted for 5 s, were measured three times with a minimum of 30 s pause between contractions [2]. The single maximum value from the three contractions for the ankle as well as the knee was then used as the respective MVIC.

Objective or target torque (TT) level was provided visually as a constant or ramp ascending torque plot. The TT was overlaid by the actual torque produced in real-time, such that both plots were displayed simultaneously on the monitor. TTs were set at constant levels of either 15% or 20% MVIC, or to a ramp ascending torque from 15–20% MVIC for each test joint. Participants were instructed to match the torque level as best they could for the duration of the 15 s test by performing isometric knee extension or ankle plantarflexion respectively. The active torque applied by the participant was displayed as a real-time visual feedback at 10 Hz, which overlaid the TT. Participants were provided 4–5 practice test repetitions to familiarise themselves with the experimental procedures. The presentation order of the signals was randomised, with all TTs (i.e. constant 15%, constant 20% and ramp of the 15−20% MVIC) presented a minimum of three times.

### Postural Sway Measurements

In order to obtain measurements of postural sway, participants were requested to stand barefoot in a quiet, bilateral stance with eyes open and with their hands by their sides. In this condition, participants focused on a visual target, positioned at eye level on the wall, approximately 3 m in front of them, and were instructed to stand as still as possible. The medial aspects of the tibial malleoli were positioned not more than 7 cm apart from one another, but on separate force platforms (AMTI OR6-7-1000, Watertown, Massachusetts, USA). In order to ensure the repeatability of the sway tests, foot locations were marked on the force platforms.

Participants were provided a minimum of 60 seconds practice before 3 repetitions of quiet standing were recorded. At least one minute relaxation was provided between each sway test. Tri-axial ground reaction force data were recorded at 120 Hz in order to allow determination of measures of the centre of pressure.

### Gait Analysis

The 3D kinematics of the right foot were measured using 4 reflective markers (14 mm) attached to the skin, tracked at 120 Hz using a 10-camera motion capture system (Vicon, OMG Ltd, Oxford, UK). Using manual palpation to locate the bone landmarks, the markers were attached to the tuber calcanei (heel), caput ossis metatarsale I (first metatarsus), caput ossis metatarsale V (fifth metatarusus) and at the base of the os metatarsale II and III (at the base of the second and the third metatarsus). Participants were requested to walk barefoot along a 10 m straight walkway, at their preferred walking speed, with recording beginning after at least 3 practice walks. A minimum of 6 walks were then measured for the determination of measures of gait.

## Data Analysis

### Force Fluctuations

All torque measurements were collected using Labview (Labview 8.6, National Instruments, Inc., USA). From each trial, the first 7 and the last 2 seconds of torque output were removed to avoid any transients during initiation or termination of the trials. All data were then low pass filtered (4^th^ order, zero-phase lag, Butterworth, 25 Hz cut-off frequency). In order to assess force fluctuations, both mean and standard deviation of the force production signal were evaluated [17]. In addition, the coefficient of variation (CV) of the force was calculated as the ratio of the standard deviation to the mean of the force output for each type of force fluctuation test and joint.

### Postural Sway

Since all force fluctuation measurements were derived from muscles that are predominantly associated with sway in the antero-posterior (A-P) direction, all tri-axial force data for postural sway were also transformed to obtain CoP times series in the A-P direction [16], with the initial and final 2 seconds of data removed to avoid boundary effects. After low-pass filtering (Butterworth, 2^nd^ order, bi-directional, 5 Hz cut-off frequency), mean (MDIST) and root mean square (RMS) distance of the CoP time series were calculated to quantify sway [22], [36] in the A−P direction. In addition, the CV of postural sway for the CoP time series in the A-P direction was calculated as the ratio of the RMS to the MDIST of sway in A-P direction.

### Gait Analysis

The trajectories of the right heel marker and the marker at the base of the second and the third metatarsus were used to extract stride time information. After low-pass filtering (Butterworth, 4^th^ order, bi-directional, 25 Hz cut-off frequency), heel strikes were identified using a foot velocity algorithm [37]. Two consecutive heel strikes were then defined as a single stride and the time elapsed between heel strikes (in seconds) provided the stride time. A minimum of 6 walks were used to calculate the variability of stride time with the first and last strides from each walk removed to avoid transients, leaving a total of approximately 30−40 strides for analysis. The CV of stride time, which represented kinematic gait variability, was calculated for each participant as the ratio of the standard deviation to the mean of the stride time for all walks.

## Statistical Analyses

### Factor Analysis for Extraction of Principal Force Fluctuation Components

Factor analysis (FA), using the “FACTOR” procedure (within the SPSS statistics package), was applied to the CV of force fluctuation obtained from the three signals (constant 15%, constant 20% MVIC and ramp 15–20% MVIC) during ankle plantarflexion and the same three signals during knee extension. The correlation analysis method was used to extract the principal components of force fluctuation before the “VARIMAX” procedure applied the appropriate rotation. Only those force fluctuation principal components (ffPCs) that had Eigenvalues greater than one then formed the dimension of the component dataset. This reduced set of ffPCs, instead of the original six CVs of force fluctuation, were considered representative of the key aspects of neuromuscular noise, and were used to compare the levels of noise between faller and non-faller cohort groupings, as well as to examine the relationships between force fluctuations, postural sway and gait variability in all subjects.

### Noise and Variability During Task Performance in Fallers and Non-fallers

In order to assess differences between faller and non-faller cohorts, non-paired t-tests were conducted on ffPCs, CV of postural sway (in the A−P direction) and CV of stride time, with significance set at 0.05.

### Relationship between Force Fluctuation, Postural Sway and Gait Variability

To examine the relationship between force fluctuations, postural sway and gait variability, two stepwise multiple linear regression (MLR) analyses were performed for each cohort, with independent variables being the derived force fluctuation components from the factor analysis. Dependent variables were firstly CV of postural sway in the antero-posterior direction, and then CV of stride time from the right limb with significance for regression analyses set at 0.05.

In the final stages of this study, we aimed to establish the relationship between force fluctuations, postural sway and gait variability. Here, since the relationship between system noise and task performance is thought to be non-linear [38], the derived components from the factor analysis were used to classify individuals into three sub-groups based on the percentiles of the distribution of the ffPCs [32], using 1^st^ and 4^th^ quartile (25^th^ –75^th^ percentile) groupings. In this approach, individuals with noise levels within 2^nd^ and 3^rd^ quartiles were termed the *nominal noise level group* (group NOM), while those outside these bounds were considered the *extreme noise level groups* (group EXT). The relationships between local noise components, postural sway and gait variability, were assessed within these sub-groups using MLR analyses.

The significance for regression analyses were set at 0.05. All statistics were conducted using the SPSS package (SPSS v20, IBM Corp., USA). Furthermore, to ensure comparability of results, all values of ffPCs, postural sway and gait variability were converted and presented as standardised *Z*-scores.

## Results

### Factor Analysis for Extraction of Principal Force Fluctuation Components

The derived dimension of the force fluctuation dataset was two, representing the key components of the original force fluctuation data (Table 1), with Eigenvalues of 2.7 and 1.2 (Table 2). The rotated component matrix (Table 2) indicated that ffPC 1 was predominantly associated with force fluctuations from the ankle plantarflexors, and this component was therefore denoted “*ankle noise*”, while ffPC 2 was almost entirely composed of force fluctuations from the knee extensors, and hence termed “*knee noise*”.

**Table 1:** Correlation coefficients for force fluctuation datasets for all participants.

	CV Ankle 15%	CV Ankle Ramp	CV Ankle 20%	CV Knee 15%	CV Knee Ramp	CV Knee 20%
**CV Ankle 15%**		**0.34** *	**0.50** *	0.25	**0.22**	**0.33** *
**CV Ankle Ramp**			**0.70** *	0.02	**0.41** *	**0.27**
**CV Ankle 20%**				**0.22**	**0.34** *	**0.28** *
**CV Knee 15%**					**0.35** *	**0.52** *
**CV Knee Ramp**						**0.32** *
**CV Knee 20%**						

Figures in bold show significance at p<0.05, while.

* indicates p<0.01.

**Table 2:** The two derived and rotated principal components (Table 1b) indicate that the first component (Eigenvalue  = 2.7) was composed of force fluctuations from the ankle plantarflexors and has thus been renamed “*ankle noise*”, while the second component (Eigenvalue  = 1.2) represented force fluctuations from the knee extensors, renamed as “*knee noise*”.

	PC 1: Ankle noise	PC 2: Knee noise
	(Eigenvalue = 2.7)	(Eigenvalue = 1.2)
**CV Ankle 15%**	**0.59**	0.32
**CV Ankle Ramp**	**0.90**	0.00
**CV Ankle 20%**	**0.88**	0.15
**CV Knee 15%**	0.00	**0.90**
**CV Knee Ramp**	**0.45**	**0.48**
**CV Knee 20%**	0.22	**0.78**

The rotated components ankle and knee noise are presented in standardised (*Z-*scores) values.

### Noise and Variability During Task Performance in Fallers and Non-fallers

Force fluctuations. The faller cohort performed the torque generation task with higher levels of *knee noise* than their non-faller counterparts (p<0.05, Table 3). However, no significant differences were observed between the faller and non-faller cohorts for *ankle noise*.

**Table 3:** Differences between faller and non-faller cohorts for components of force fluctuations, Ankle and Knee noise, postural sway, and gait variability.

		Non-fallers (N = 36)	Fallers (N = 34)
**Force fluctuations**	Ankle noise	0.01 (±0.90)	−0.1 (±1.11)
	Knee noise	−**0.22 (±0.74)**	**0.23 (±1.2)**
**Postural sway**	CV Sway A-P	−**0.25 (±0.69)**	**0.26 (±1.2)**
**Gait variability**	CV Stride time	−0.32 (±0.90)*	0.34 (±1.0)*

Force fluctuations were quantified using the coefficient of variation (CV), of the force production signals with TTs set at 15%, 20% and 15–20% ramp for ankle plantarflexors and knee extensors. CV of postural sway in the A-P direction was evaluated as the ratio of the RMS of sway to the mean distance of sway in the A-P direction. Finally, gait variability was quantified using CV of stride time during walking from the right leg. Values in bold represent significance with p<0.05, while * represents significance at p<0.01.

#### Postural sway

During quiet standing, faller cohorts exhibited significantly higher values for CV of sway in the antero-posterior direction compared to the non-fallers (p = 0.02, Table 3).

#### Gait variability

The CV of stride time was approximately 44% higher for the fallers compared to the non-fallers (p = 0.03, Table 3).

### Relationship between Force Fluctuation, Postural Sway and Gait Variability

**Figure 1:**
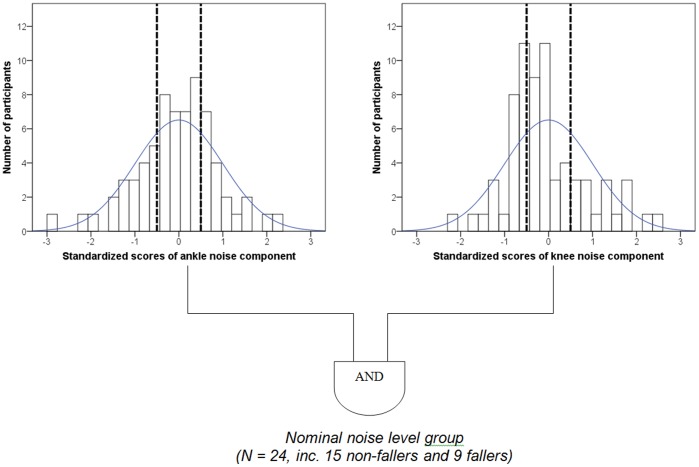
Classification of participants according to neuromuscular noise. Histogram of the 1^st^ and 2^nd^ rotated components obtained using the factor analysis, representing the ankle and knee force fluctuations, or “*ankle noise*” and “*knee noise*” respectively. The dotted lines represent the 25^th^ and the 75^th^ percentile boundaries. The bell shaped curve illustrates the normal distribution plot for the knee as well as the ankle noise components. The participants that had both ankle noise and knee noise values inside the dotted lines (25–75^th^) were classified in the nominal noise level group (group NOM, N = 24, inc. 15 non-fallers and 9 fallers), while those that had values outside the dotted lines formed the extreme noise level group (group EXT).

**Figure 2:**
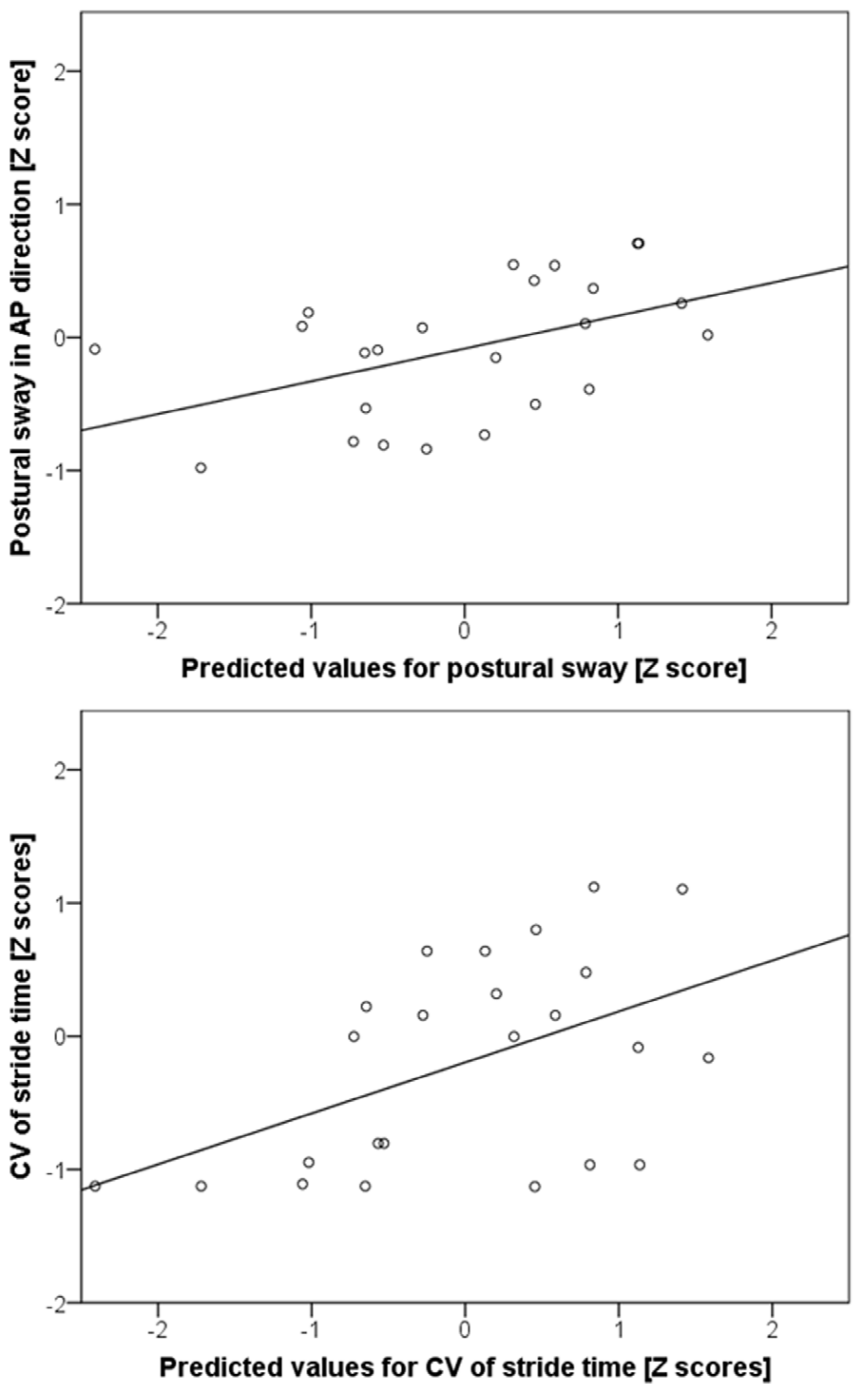
Relationship between neuromuscular noise and task variability. The regression plots for group NOM using standardised Z-scores of the measured postural sway in AP direction (Figure 2; Top) and stride time variability (Figure 2; Bottom) represented on the y-axis against standardised Z-scores of the predicted values from the regression. Independent variables are ankle and knee noise. The r^2^ for the regression with postural sway in A-P direction as dependent variable was 0.23 and with stride time variability was 0.24.

MLR analyses found no association for fallers or non-fallers between *ankle* and *knee noise*, and postural sway or gait variability. However, classification (Figure 1) into *nominal* (group NOM, 24 participants aged 68±5 years) and *extreme noise level groups* (group EXT, 46 participants, aged 70±5 years), revealed a non-linear relationship between noise and task performance (Figure 2) as follows:

In group NOM, there was a significant relationship between *knee noise* and CV of postural sway in Z-score values (r^2^ = 0.23, *p = *0.02, Figure 2 top).
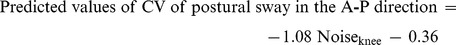
where Noise_knee_ represents *knee noise* (Tables 1 & 2).

Similarly, a significant relationship was observed between *knee noise* and CV of stride time in Z-scores, (r^2^ = 0.24, *p = *0.01, Figure 2 bottom).
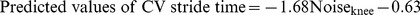



Subjects in group EXT (21 participants from the non-faller and 25 from the faller groupings), tended to be older than the participants of group NOM (*p = *0.06). MLR analyses revealed no relationships between either *ankle* or *knee noise*, and postural sway or gait variability. Furthermore, group EXT had lower levels of MVIC in the knee extensors (*p = *0.04) and tended to walk slower (*p = *0.08) with shorter strides (*p = *0.01) than group NOM.

## Discussion

Variability during task performance is known to be increased in subjects who have fallen [12], [31], [39], but the aetiology of variability during static and dynamic functional task performance has until now, remained unclear. Based on the notion that the fluctuations during isometric force production tasks closely represent neuromuscular noise [2], this study examined whether levels of neuromuscular noise could predict variability during standing and walking. In this study we therefore investigated the levels of, as well as the relationships between, neuromuscular noise in the knee extensors and ankle plantar-flexors, and variability during task performance in cohorts of fallers and non-fallers. The results of this study indicate that fallers possess higher levels of noise during muscular force production in the knee extensors (p<0.05) but not in the ankle plantarflexors. In addition, fallers also exhibited higher levels of postural sway and gait variability than their non-faller counterparts. We observed that subjects with nominal levels of neuromuscular noise throughout the lower extremities (group NOM) exhibited a clear association (r^2^>0.23), whereas those with extreme (high as well as low) noise levels (group EXT) did not. In addition, group EXT possessed lower stride lengths, isometric muscle strengths and walking speeds. This suggests that extreme levels of noise during force production in the lower extremities are associated with functional deficits consistent with reduced gait stability [12], [26], [39] and even falling [22], [23], [31], [40], and could therefore be considered a key neuromuscular deficit in the elderly.

Excessive levels of variability during standing as well as walking are factors known to limit functional performance in the elderly [14], [26], [27], [31], [32], [41]. However, recent studies indicate that *nominal* levels of variability might be essential for effective task learning [28]–[30], while *extreme* levels of variability, both high and low, might indicate motor-related pathologies [14], [22], [24], [26], [31], [32], [38]. Our premise in this study was that neuromuscular noise is responsible for the variability in output task performance. This was demonstrated in our study by a clear relationship between noise in the knee extensors and variability during standing and walking in subjects with nominal levels of neuromuscular noise. However, no such relationships were present in subjects who exhibited excessively high or extremely low levels of noise, suggesting either some compensation mechanisms or neuromuscular deficits. Such adaptations might well be due to a remodelling of the sensori-motor system in these individuals, possibly also leading to the observed lower levels of isometric strength in the knee extensors and shorter strides, as well as a tendency towards slower walking.

In order to avoid excessive levels of variability that could lead the system either to operate closer to or even outside the limits of stability during continuous standing or walking, the human sensori-motor control system attempts to regulate and maintain the centre of mass within the base of support by using a variety of strategies [18], [20], [21], [23], [34], [40], [42]–[44]. The results of this study suggest not only that the quality of control of posture and movement, but also the choice of control strategy seems to be influenced by the level of noise in the sensori-motor system. Since fluctuations during force production at the knee were a predictor of sway and gait variability in group NOM, it seems that one mechanism for effective task performance, at least in these subjects, might well have been a strategy involving optimisation of neuromuscular noise in the force outputs [11], [15]. While the exact mechanisms remain unclear, it appears that effective regulation of neuromuscular noise within the sensori-motor system might be a prerequisite for improved task performance.

Fluctuations in force generation during submaximal isometric contractions within a particular muscle are known to be dependent upon the discharge properties of the motor units recruited to perform the required task [1], [3]. However, variability during task performance such as standing or walking, depends not only the recruitment of motor units within a particular muscle, but also on other factors such as muscle force-length and force-velocity relationships [45], muscle coordination [46], sensori-motor feedback quality [47], and alternating activation of agonist, synergists and antagonists [1], [13], [18], [21], [35], [39], [44], as well as the requirements of the task itself [6], [13], [48]. For example, task performance during position-matching (anisometric) activities is thought to differ from performance during position-maintaining (isometric) tasks [6], [13], [48]. While many of these complexities could not be specifically considered within our study, the underlying mechanisms governing the variability of output task performance seem to be closely associated with the levels of neuromuscular noise. Further support for such a concept comes from studies that show a clear relationship between the variability in discharge properties of active motor units, and the task performance, including the variability of force output [6], [13], [48]. Furthermore, it is important to note that in this study, fluctuations were measured at 15–20% MVIC, but it is possible that fluctuations at different recruitment levels might also play a role in sway during standing [49], as well as in variability during walking. It is therefore plausible that assessment of different aspects of neuromuscular noise, including different contractions as well as at varying force levels, might allow a more comprehensive understanding of the relationships between neuromuscular noise levels and task performance. On the other hand, according to the Optimal Control Theory proposed by Harris and Wolpert [11], [15], which suggests that SD and mean of the produced force are proportional, the relationship between neuromuscular noise levels and variability during task performance should be independent of the force production levels. Further investigation is therefore required in order to provide a deeper understanding of the *in vivo* relationships between force levels, the recruitment of motor units, variability of discharge rates of the active motor neurons, and the quality of task performance [13].

While a variety of both clinical and functional approaches have been used to investigate the risk of falling in the elderly (for an overview see [31], [50]), we have addressed the role of the underlying physiological characteristics, specifically neuromuscular noise, on task performance in these populations. In this study, we examined the relationships between force fluctuations, postural stability and gait variability in faller and non-faller cohorts. Elderly fallers are known to possess higher levels of sway [22], as well as increased gait variability [24]–[26], [31], than their non-falling counterparts, and this has been confirmed in our cohorts (Table 3). In this study, fluctuations during force production were larger in elderly fallers compared to non-fallers and this deficit might have affected the control of both the timing of stride events during walking as well as sway during standing. More importantly, for the first time, it was demonstrated in this study that neuromuscular noise levels might play a direct role in the quality of both static and dynamic functional task performance. Specifically, individuals with nominal levels of noise exhibited a clear association between neuromuscular noise and variability during task performance. On the other hand, individuals with extreme levels of noise, did not exhibit any such relationship, but also tended to be older and walked slower, while having lower isometric strength in the knee extensors and smaller stride lengths. Although further investigation is required, it seems reasonable that an assessment of force fluctuations in the musculature of the lower limb, achievable in a clinical setting, could contribute towards the early identification of motor related pathologies. Whether intervention programmes or clinical therapies to improve muscular control and steadiness are then able to also reduce e.g. a subject’s risk of falling, remains to be elucidated.

### Conclusions

Individuals with nominal levels of noise exhibited a clear association between neuromuscular noise, assessed as force fluctuations from muscles of the lower extremity, and the variability in performing both static and dynamic functional tasks. However, in individuals with extreme levels of neuromuscular noise, no such relationships were observed, and these subjects possessed neuromuscular compensations such as lower stride length and isometric strength. The results of this study therefore suggest that extreme levels of neuromuscular noise, both excessively high and low, constitute a key functional deficit in elderly individuals.
